# Grindstone Chemistry: Design, One-Pot Synthesis, and Promising Anticancer Activity of Spiro[acridine-9,2′-indoline]-1,3,8-trione Derivatives against the MCF-7 Cancer Cell Line

**DOI:** 10.3390/molecules25245862

**Published:** 2020-12-11

**Authors:** Perumal Gobinath, Ponnusamy Packialakshmi, Ali Daoud, Saud Alarifi, Akbar Idhayadhulla, Surendrakumar Radhakrishnan

**Affiliations:** 1 Department of Chemistry, Nehru Memorial College, Affiliated Bharathidasan University, Puthanamapatti, Tamilnadu 621007, India; gopi10207005@gmail.com (P.G.); packiyaponnusamy96@gmail.com (P.P.); idhayadhulla@nmc.ac.in (A.I.); 2 Department of Zoology, College of Sciences, King Saud University (KSU), P.O. Box 2455, Riyadh 11451, Saudi Arabia; aalidaoud@ksu.edu.sa (A.D.); salarifi@ksu.edu.sa (S.A.)

**Keywords:** isatin, indole, solvent-free conditions, cyclocondensation, grinding, p-TSA catalyst, anticancer activity

## Abstract

In this study, the synthesis of one-pot 10-phenyl-3,4,6,7-tetrahydro-1*H*-spiro [acridine-9,2′-indoline]-1,3,8-trione derivatives was achieved via a four-component cyclocondensation reaction, which was carried out in solvent-free conditions, and using p-toluenesulfonic acid (p-TSA) as a catalyst. The product was confirmed by FT-IR, ^1^H-NMR, ^13^C-NMR, mass spectra, and elemental analysis. Furthermore, the anticancer activity was screened for all compounds. Among these compounds, compound **1c** was more effective (GI_50_ 0.01 µm) against MCF-7 cancer cell lines than standard and other compounds. Therefore, the objective of this study was achieved with a few promising molecules having been demonstrated to be potential anticancer agents.

## 1. Introduction

**Figure 1:**
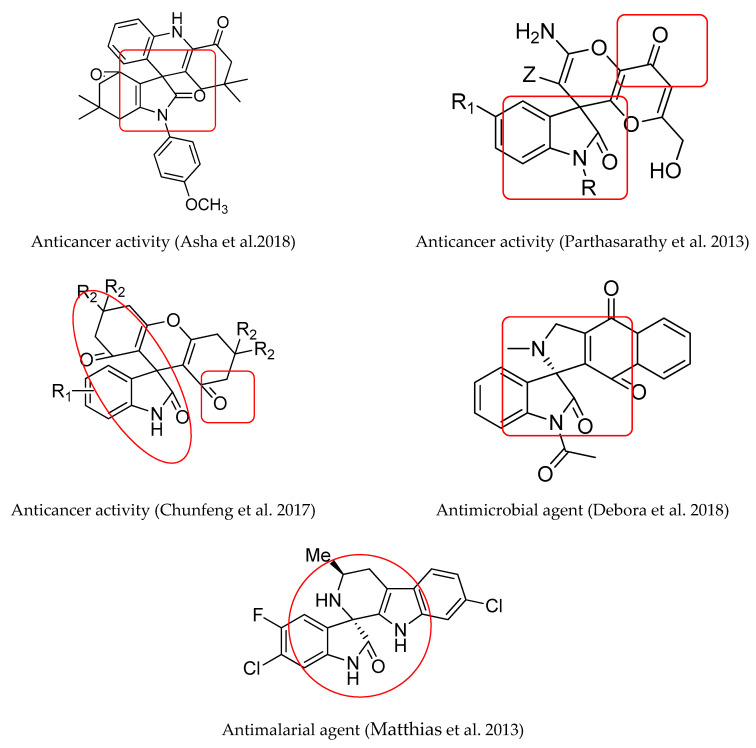
Some medicinally important compounds with spiroindole acridine derivatives [30,31,32,33,34].

The advantages of acridine as a helpful nucleus in medicinal chemistry has consequently led to new designs, and the growth of its bi- and tri-analogues for delivering superior effects in targeted therapy compared to mono-analogues. Additionally, at present isatin and its derivatives may also be the most helpful beginning resources or ancestors within the synthesis of a good variety of spirocyclic indoles [1]. The spiro oxindole may be an advantageous heterocyclic core present in a sizable number of novel inhibitors with microtubule assembly [2], whereas pteropodines act as positive modulators of muscarinic M2 and 5-HT2 receptors [3]. Moreover, the spiro oxindole system is also a nucleus scaffold of many non-natural pharmaceutical elements with a wide assortment of biological uses, such as inhibitors of the human NK-1 receptor [4], antimicrobials [5], antineoplastics [6], and antibiotics [7]. Primarily, anticancer potency, as well as a number of other factors such as topoisomerase and telomerase inhibition, initiation of ROS mediated oxidative stress, cell cycle arrest, and interaction with P-glycoprotein, also come into play which makes the acridine/acridone moiety a privileged scaffold for anticancer chemotherapy [8]. The acridines exhibit antiprotozoal [9], antihelmintic [10] antineoplastic [11] and antiviral [12] activities. The ‘Grindstone Chemistry’ technique has been used as an inexperienced and speedy methodology for the synthesis of organic compounds [13,14]. Grindstone Chemistry may involve a small alteration and has established that several reactions are often carried out in high yields by grinding two or more solids [15]. The formation of 10-phenyl-3,4,6,7-tetrahydro-1*H*-spiro [acridine-9,2′-indoline]-1,3,8-trione as a by-product is a part of our program aimed at the preparation of heterocyclic compounds [16,17,18,19,20,21,22,23,24], particularly spirooxindoles [25,26,27,28,29]. Some spiroindoles exhibit medicinally important activity, as shown in Figure 1. In Grindstone Chemistry, reactions are often performed by grinding the reactants for many minutes, without using any organic solvent. Apparently, this methodology is very effective for endothermic reactions. Based on the above results, we prepared new 10-phenyl-3,4,6,7–tetra hydro-1*H*-spiro [acridine-9,2-indolidine]-1,3,8-trione derivatives via Grindstone Chemistry and the new compounds were screened for anticancer activity.

## 2. Results and Discussion

### 2.1. Chemistry

**Scheme 1:**
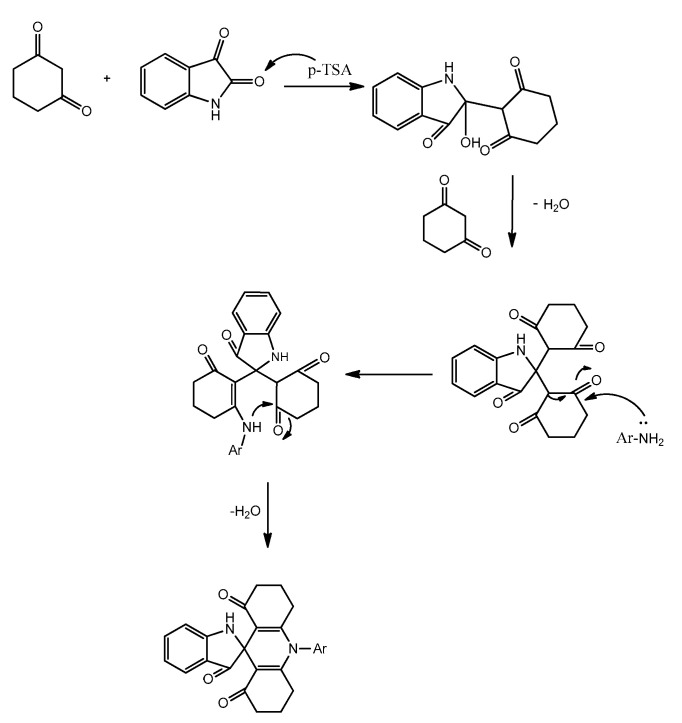
Proposed mechanism for the synthesis of spiro [acridine-9,2′-indoline]-1,3,8-trione.

**Scheme 2:**
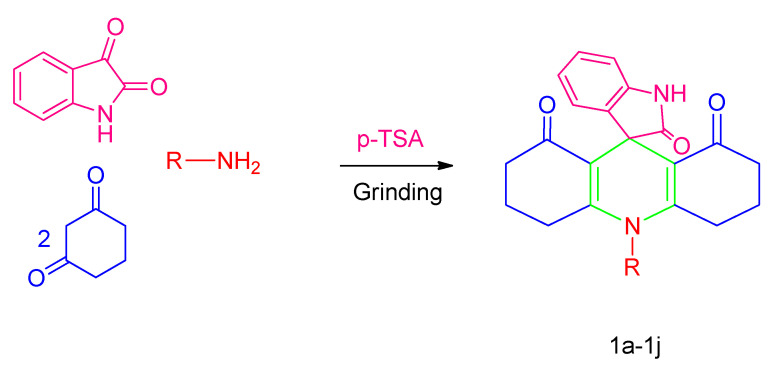
One-pot synthesis of spiro[acridine-9,2′-indoline]-1,3,8-trione derivatives.

The synthetic methodology was evaluated with isatins, substituted anilines, and 1,3-cyclohexanedione, and the procedure followed that of [35]. All synthesized compounds were characterized by IR, ^1^H and ^13^C-NMR analysis. The IR spectrum of the compounds (**1a**–**1j**) showed an absorption band at 3258 to 3500 cm^−1^ due to N–H stretching, an absorption band at 2922 to 3234 cm^−1^ due to Ar–H stretching, an absorption band at 1664 to 1716 cm^−1^ due to C=O stretching, and another absorption band at 1276 to 1516 cm^−1^ due to C–N stretching. Compound 1h exhibited an absorption band for the Cl–C group at 799 cm^−1^ and compounds **1b**, **1f** and **1i** displayed an absorption band for the NO_2_–C group at 1504–1523 cm^−1^. The ^1^H NMR spectrum of compounds **1a**–**1j** showed a singlet at δ 10.10 to 10.26, attributable to N–H protons present in the isatin ring. The ^13^C-NMR spectrum of compounds **1a**–**1j** exhibited peaks at δ 126.9 to 127.8, corresponding to the 2- position of C in the isatin ring. The corresponding spiro-[acridine-9,2′-indoline]-1,3,8-trione **1a**–**1j** was obtained in smart yields in similar conditions, which are shown in Table 1. We have not established an exact mechanism for the formation of spiro[acridine-9,2′-indoline]-1,3,8-trione derivatives, however, a sensible possibility is shown in Scheme 1. This scheme supposes that the initial addition of 1,3-cyclohexanedione to the isatin yielded the intermediate, which then reacted with an extra molecule of 1,3-cyclohexanedione. Finally, adding the substituted aniline to the intermediate, followed by cyclization, afforded the product **1a**–**1j**. Scheme 2 indicates the synthesis of spiro acridine derivatives of **1a**-**1j**.

#### Anticancer Activity

The compounds (**1a**–**1j**) were evaluated for anticancer activity. The MCF-7 cancer cell line was used to test all compounds at a cytotoxic assay dose of 100 µM at 48 h (MTT anticancer assay measuring the functionality of animal and human cells) Table 2 indicates the results of each test compound with the growth inhibitor concentration (GI_50_), total growth of inhibition (TGI), and lethal concentration (LC_50_) values. The cytotoxic effect of **1c** (GI_50_ = 0.01 µM) was highly active, while **1b** (GI_50_ = 0.02 µm) was substantially active, and 1i (GI_50_ = 0.03 µM) and **1j** (GI_50_ = 0.04 µM) were reasonably active in the MCF-7 cell line. Other compounds also exhibited significant activity. The cytotoxicity values are presented in Table 2.

## 3. Experimental

All of the chemicals were synthetic grade and commercially purchased from Merck. The melting point was determined by an open capillary tube and it was uncorrected. The IR spectra were recorded in KBr on a Shimadzu 8201pc (4000–400 cm^−1^). ^1^H and ^13^C-NMR spectra were recorded on a Bruker Avance II NMR spectrometer 300 MHz with DMSO-d_6_ as the solvent, using tetramethylsilane (TMS) as an internal standard. Mass spectra (Perkin Elmer) and the elemental analysis (C, H, and N) were recorded using an elemental analyzer model (Varian EL III).

### 3.1. Synthesis of Compounds **1a**–**1j**

A mixture of isatin (10 mmol), phenylamine (10 mmol), and 1,3-cyclohexanedione (20 mmol), as well as p-toluenesulfonic acid (p-TSA) as a catalyst, were ground for 3–4 min, leading to a red color of 10-phenyl-3,4,6,7-tetrahydro-1*H*-spiro[acridine-9,2′-indoline]-1,3,8-trione. The final product was washed with water and recrystallized with ethanol. The purity of the compound was checked by TLC. Hexane was used as eluting solvent in TLC. The product was separated by column chromatography. All the compounds were recrystallized by ethanol (see Supplementary Materials).

*1.0-phenyl-3,4,6,7-tetrahydro-spiro[acridine-9,3-indoline]-1,2,8-trione* (**1a**): Brown powder; mp 108–110 °C; IR: 3334 (NH), 3234 (Ar-H),1716 (C=O), 1448 (C-N); ^1^H NMR (CDCl_3_, 500 MHz): δ = 10.06 (s, 1H,NH), 7.34–6.81 (m, 4H, N-Ph), 7.30–7.07 (m, 4H, Ph-isatin), 3.16 (m, 4H, CHD), 2.82 (m, 4H, CHD), 1.67 (dd, 4H, J= 7.5, 1.5 Hz, CHD); ^13^C-NMR (CDCl_3_, 125 MHz): 168.2, 153.3, 141.2, 141.1, 129.6, 129.5, 127.8, 124.8, 122.8, 122.4, 115.2, 108.2, 51.3, 36.0, 21.3, 18.7; EI-MS: 411 (M^+^, 29.7%); Analysis calculated for C_26_H_22_N_2_O_3_: C, 76.08; H, 5.40; Found: C, 76.06; H, 5.39.

*10-(4-nitrophenyl)-3,4,6,7-tetrahydro-1H-spiro[acridine-9,3’-indoline]1,2’,8(2H,5H,10H)-trione* (**1b**): Yellow needles; mp 108–110 °C; IR: 3350 (NH), 2920 (Ar-H), 1680 (C=O), 1516 (C-N), 1523 (NO_2_); ^1^H NMR (CDCl_3_,500 MHz): δ = 10.11 (s, 1H, NH), 8.01–6.62 (m, 4H, N-Ph), 7.31–7.09 (m, 4H, Ph (isatin)), 3.18 (m, 4H, CHD), 2.83 (m, 4H, CHD), 1.68 (dd, 4H, *J* = 7.5, 1.5 Hz, CHD); ^13^C-NMR (CDCl_3_, 125 MHz): 168.3, 153.4, 147.3, 141.2, 137.9, 129.7, 127.9, 124.7, 123.9, 115.3, 108.3, 51.4, 36.1, 21.4, 18.8; EI-MS: 455 (M^+^, 29.7%); Analysis calculated for C_26_H_21_N_3_O_5;_ C, 68.56; H, 4.56; Found: C, 68.54; H, 4.53.

*10-(4-methoxyphenyl)-3,4,6,7-tetrahydro-1H-spiro[acridine-9,3’-indoline]-1,2’,8(2H,5H, 10H)-trione* (**1c**): Yellow needles; mp 108–110 °C; IR: 3425 (NH), 2922 (Ar-H), 1664 (C=O), 1452 (C-N); ^1^H NMR (CDCl_3_,500 MHz): δ = 10.12 (s, 1H, NH), 7.32−7.10 (m, 4H, Ph (isatin)), 6.74−6.00 (m, 4H, N-Ph), 3.83 (s, 3H, -OCH_3_), 3.19 (m, 4H, CHD), 2.84(dd, 4H, *J* = 7.5, 1.5 Hz, CHD); ^13^C-NMR (CDCl_3_, 125 MHz): 168.4, 153.3, 141.3, 133.5, 129.8, 127.6,123.5, 115.4, 115.1, 108.4, 55.8, 51.5, 36.2, 21.5, 18.8; EI-MS: 441 (M^+^, 30.4%); Elemental analysis calculated value for C_27_H_24_N_2_O_4;_ C, 73.62; H, 5.49; Found: C, 73.61; H, 5.48.

*10-(2-methoxyphenyl)-3,4,6,7-tetrahydro-1H-spiro[acridine-9,3’-indoline]-1,2’,8(2H,5H, 10H)-trione* (**1d**): Yellow needles; mp 108–110 °C; IR: 3456 (NH), 3112 (Ar-H), 1709 (C=O), 1336 (C-N); ^1^H NMR (CDCl_3_,500 MHz): δ = 10.14 (s, 1H, NH), 7.33−7.12 (m, 4H, Ph (isatin)), 6.85−6.12 (m, 4H, N-Ph), 3.83 (s, 3H, -OCH_3_), 3.20 (m, 4H, CHD), 2.85 (dd, 4H, *J* = 7.5, 1.5 Hz, CHD); ^13^C-NMR (CDCl_3_, 125 MHz): 168.5, 153.5, 148.9, 141.0, 129.5, 128.2, 127.8, 127.5, 125.2, 122.6, 115.2, 110.1, 108.3, 55.8, 51.6, 36.3, 21.6, 18.9; EI-MS: 441 (M^+^, 30.4%); Analysis calculated value for C_27_H_24_N_2_O_4;_ C, 73.62; H, 5.49; Found: C, 73.61; H, 5.48.

*10-(m-tolyl)-3,4,6,7-tetrahydro-1H-spiro[acridine-9,3’-indoline]-1,2’,8(2H,5H,10H)-trione* (**1e**): Yellow needles; mp 108–110 °C; IR: 3500 (NH), 3096 (Ar-H), 1695 (C=O), 1276 (C-N); ^1^H NMR (CDCl_3_, 500 MHz): δ = 10.16 (s, 1H, NH), 7.34−7.13 (m, 4H, Ph (isatin)), 7.08−5.70 (m, 4H, N-Ph), 3.21 (m, 4H, CHD), 2.88 (dd, 4H, *J* = 7.5, 1.5 Hz, CHD), 2.34 (s, 3H, CH_3_); ^13^C-NMR (CDCl_3_, 125 MHz): 168.6, 153.6, 141.1, 129.6, 129.4, 127.4, 124.2, 119.8, 119.0, 115.3, 108.4, 51.7, 36.4, 21.7, 19.0; EI-MS: 425 (M^+^, 30.3%); Elemental analysis calculated value for C_27_H_24_N_2_O_3;_ C, 76.39; H, 5.70; Found: C, 76.37; H, 5.68.

*10-(2-nitrophenyl)-3,4,6,7-tetrahydro-1H-spiro[acridine-9,3’-indoline]-1,2’,8(2H,5H,10H)-trione* (**1f**): Yellow needles; mp 108–110 °C; IR: 3425 (NH), 2922 (Ar-H), 1664 (C=O), 1452 (C-N), 1504 (NO_2_); ^1^H NMR (CDCl_3_,500 MHz): δ = 10.18 (s, 1H, NH), 8.01−6.89 (m, 4H, N-Ph), 7.35−7.14 (m, 4H, Ph (isatin)), 3.22 (m, 4H, CHD), 2.86 (dd, 4H, *J* = 7.5, 1.5Hz, CHD); ^13^C-NMR (CDCl_3_, 125 MHz): 168.7, 153.7, 147.8, 134.6, 130.5, 129.9, 129.3, 127.3, 126.6, 124.8, 115.4, 108.6, 51.8, 36.5, 21.8, 19.2; EI-MS: 456 (M^+^, 29.7%);Elemental analysis calculated value for C_26_H_21_N_3_O_5;_ C, 68.56; H, 4.65; Found: C, 68.54; H, 4.64.

*10-(p-tolyl)-3,4,6,7-tetrahydro-1H-spiro[acridine-9,3’-indoline]-1,2’,8(2H,5H,10H)-trione* (**1g**): Yellow needles; mp 108–110 °C; IR: 3221 (NH), 3029 (Ar-H), 1703 (C=O), 1276 (C-N); ^1^H NMR (CDCl_3_, 500 MHz): δ = 10.20 (s, 1H, NH), 7.36–7.15 (m, 4H, Ph (isatin)), 6.98–6.08 (m, 4H, N-Ph), 3.23 (m, 4H, CHD), 2.87 (dd, 4H, *J* = 7.5, 1.5 Hz, CHD), 2.34 (s,3H, CH_3_); ^13^C-NMR (CDCl_3_, 125 MHz): 168.8, 153.8, 138.2, 131.2, 129.8, 129.2, 127.2, 123.2, 115.6, 108.7, 51.9, 36.6, 21.9, 19.3; EI-MS: 425 (M^+^, 30.3%); Elemental analysis calculated value for C_27_H_24_N_2_O_3;_ C, 76.39; H, 5.70; Found: C, 76.37; H, 5.67.

*10-(2-chlorophenyl)-3,4,6,7-tetrahydro-1H-spiro[acridine-9,3’-indoline]-1,2’,8(2H,5H,10H)-trione* (**1h**): Yellow needles; mp 108–110 °C; IR: 3258 (NH), 3012 (Ar-H), 1695 (C=O), 1452 (C-N), 799 (Cl); ^1^H NMR (CDCl_3_, 500 MHz): δ = 10.22 (s, 1H, NH),7.40–6.57 (m, 4H, N-Ph), 7.37–7.16 (m, 4H, Ph (isatin)), 3.24 (m, 4H, CHD), 2.88 (dd, 4H, *J* = 7.5, 1.5 Hz, CHD); ^13^C-NMR (CDCl_3_, 125 MHz): 168.9, 153.9, 144.2, 132.2, 130.7, 29.1, 127.1, 127.6, 125.4,123.8, 115.7, 108.8, 52.0, 36.7, 22.0, 19.4; EI-MS: 446 (M^+^, 36.7%); Elemental analysis calculated value for C_26_H_21_N_2_O_3;_ C, 70.19; H,4.76; Found: C, 70.17; H, 4.74.

*10-(2,4-dinirophenyl)-3,4,6,7-tetrahydro-1H-spiro[acridine-9,3’-indoline]-1,2’,8(2H,5H, 10H)-trione* (**1i**): Yellow needles; mp 108–110 °C; IR: 3350 (NH), 3027 (Ar-H), 1664 (C=O), 1452 (C-N), 1516 (NO_2_); ^1^H NMR (CDCl_3_,500 MHz): δ = 10.24 (s, 1H, NH), 8.85–7.15 (m, 4H, N-Ph), 7.38–7.17 (m, 4H, Ph (isatin)), 3.25 (m, 4H, CHD), 2.89 (dd, 4H, *J* = 7.5, 1.5 Hz, CHD); ^13^C-NMR (CDCl_3_, 125 MHz): 167.0, 154.0, 147.9, 138.8, 138.0, 130.8, 129.2, 127.0, 120.8, 118.1, 115.8, 108.9, 52.3, 36.8, 22.1, 19.5; EI-MS: 501 (M^+^, 30.1%); Elemental analysis calculated value for C_26_H_20_N_4_O_7;_ C, 62.40; H, 4.03; Found: C, 62.38; H, 4.01.

*10-(4-bromophenyl)-3,4,6,7-tetrahydro-1H-spiro[acridine-9,3’-indoline]-1,2’,8(2H,5H,10H)-trione* (**1j**): Yellow needles; mp 108–110 °C; IR: 3425 (NH), 2922 (Ar-H), 1664 (C=O), 1452 (C-N); ^1^H NMR (CDCl_3_,500 MHz): δ = 10.26 (s,1H, NH), 7.54–7.35 (m, 4H, N-Ph), 7.39–7.18 (m, 4H, Ph (isatin)), 3.26 (m, 4H, CHD),2.90 (dd, 4H, *J* = 7.5, 1.5Hz,CHD); ^13^C-NMR (CDCl_3_, 125 MHz): 167.1, 154.2, 140.2, 130.1, 129.5, 125.4,126.9, 116.7, 115.9, 109.1, 52.4, 36.9, 22.3, 19.6; EI-MS: 490 (M^+^, 28.6%); Elemental analysis calculated value for C_26_H_21_N_2_O_3;_ C, 70.19; H, 4.76; Found: C, 70.17; H, 4.74.

### 3.2. Cytotoxic Activity

The newly synthesized compounds (**1a**–**1j**) were screened for their cytotoxic activity according to the procedure suggested in previous literature [36].

## 4. Conclusions

We have reported a one-pot, and four-component methodology for the synthesis of 10-phenyl-3,4,6,7-tetrahydro-1*H*-spiro[acridine-9,2-indolidine]-1,3,8-trione derivatives via Grindstone Chemistry. The cytotoxic activity was screened for all compounds, demonstrating that compound 1c was highly active (GI_50_ 0.01 µm) against MCF-7 cancer cell lines in comparison to doxorubicin and other compounds. In conclusion, the synthesized acridine-based indole derivatives can be considered as potential therapeutic lead molecules for anticancer activity. 

## Figures and Tables

**Table 1:** Optimization of the reaction conditions for compounds (**1a**–**1j).**

Compounds	R	Time (Min)	Yield (%)
**1a**	Ph	10	92
**1b**	-4-NO_2_-C_6_H_4_	10	91
**1c**	-4-OCH_3_-C_6_H_4_	10	88
**1d**	-2-OCH_3_-C_6_H_4_	10	87
**1e**	-3-CH_3_-C_6_H_4_	10	95
**1f**	-2-NO_2_-C_6_H_4_	10	82
**1g**	-4-CH_3_-C_6_H_4_	10	94
**1h**	-2-Cl-C_6_H_4_	10	87
**1i**	-2,4-NO_2_-C_6_H_4_	10	78
**1j**	-4-Br-C_6_H_4_	10	91

**Table 2:** Anticancer activity of compounds (**1a**–**1j**).

Compounds	MCF-7 Cell Line		
	GI_50_ (µM)	TGI (µM)	LC_50_ (µM)
**1a**	02.7 ± 0.11	06.5 ± 1.06	08.1 ± 0.02
**1b**	0.02 ± 0.29	0.41 ± 2.32	0.87 ± 0.24
**1c**	0.01 ± 0.34	0.02± 1.19	0.71 ± 1.21
**1d**	0.09 ± 0.61	0.55 ± 0.38	1.40 ± 1.15
**1e**	0.19 ± 0.93	0.20 ± 1.32	0.70 ± 1.17
**1f**	0.52 ± 0.09	10.1 ± 2.22	22.1 ± 0.12
**1g**	0.35 ± 0.83	0.66 ± 1.12	14.2 ± 0.82
**1h**	0.26 ± 0.14	04.4 ± 1.09	09.7 ± 0.28
**1i**	0.03 ± 0.49	0.69 ± 0.61	2.12 ± 0.21
**1j**	0.04 ± 0.34	0.34 ± 0.55	1.01 ± 0.18
**Doxorubicin**	0.02 ± 0. 70	0.21 ± 0. 19	0.74 ± 0.35

## Supplementary Materials

The following are available online, 1H-NMR and 13C-NMR Spectrum of the compounds **1a**–**1j**.
