# Correction: Gobinath, P., et al. Grindstone Chemistry: Design, One-Pot Synthesis, and Promising Anticancer Activity of Spiro[acridine-9,2′-indoline]-1,3,8-trione Derivatives against the MCF-7 Cancer Cell Line. *Molecules* 2020, *25*, 5862

**DOI:** 10.3390/molecules26041131

**Published:** 2021-02-20

**Authors:** Perumal Gobinath, Ponnusamy Packialakshmi, Ali Daoud, Saud Alarifi, Akbar Idhayadhulla, Radhakrishnan Surendrakumar

**Affiliations:** 1 Department of Chemistry, Nehru Memorial College, Affiliated Bharathidasan University, Puthanamapatti, Tamilnadu 621007, India; gopi10207005@gmail.com (P.G.); packiyaponnusamy96@gmail.com (P.P.); idhayadhulla@nmc.ac.in (A.I.); 2 Department of Zoology, College of Sciences, King Saud University (KSU), P.O. Box 2455, Riyadh 11451, Saudi Arabia; aalidaoud@ksu.edu.sa (A.D.); salarifi@ksu.edu.sa (S.A.)

In the original article [1], there was a mistake in Scheme 1 as published. The original Scheme 1 was:

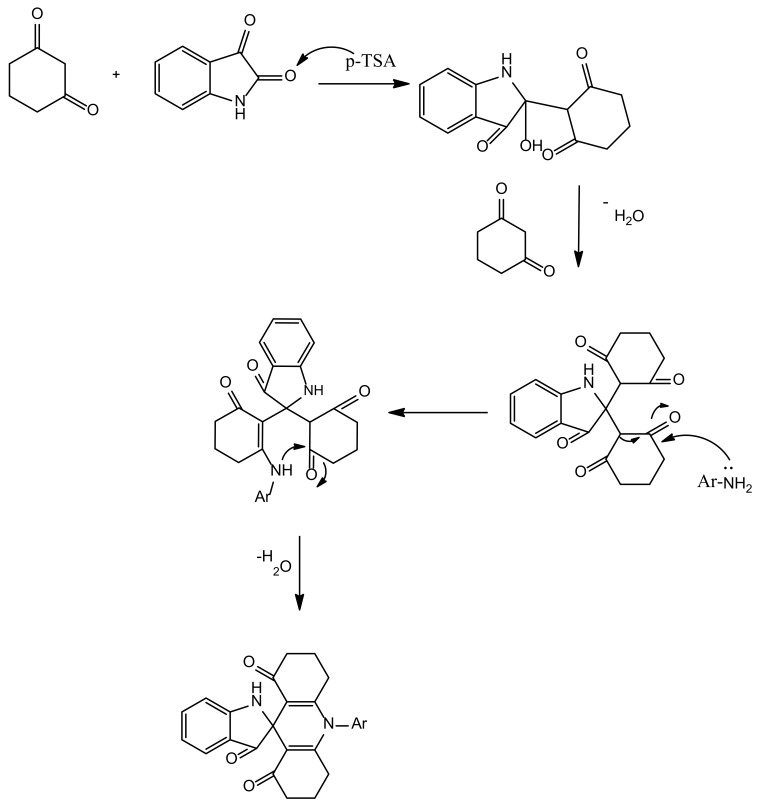



The corrected Scheme 1 appears below. The authors apologize for any inconvenience caused and state that the scientific conclusions are unaffected. The original article has been updated.

## Figures and Tables

**Scheme 1:**
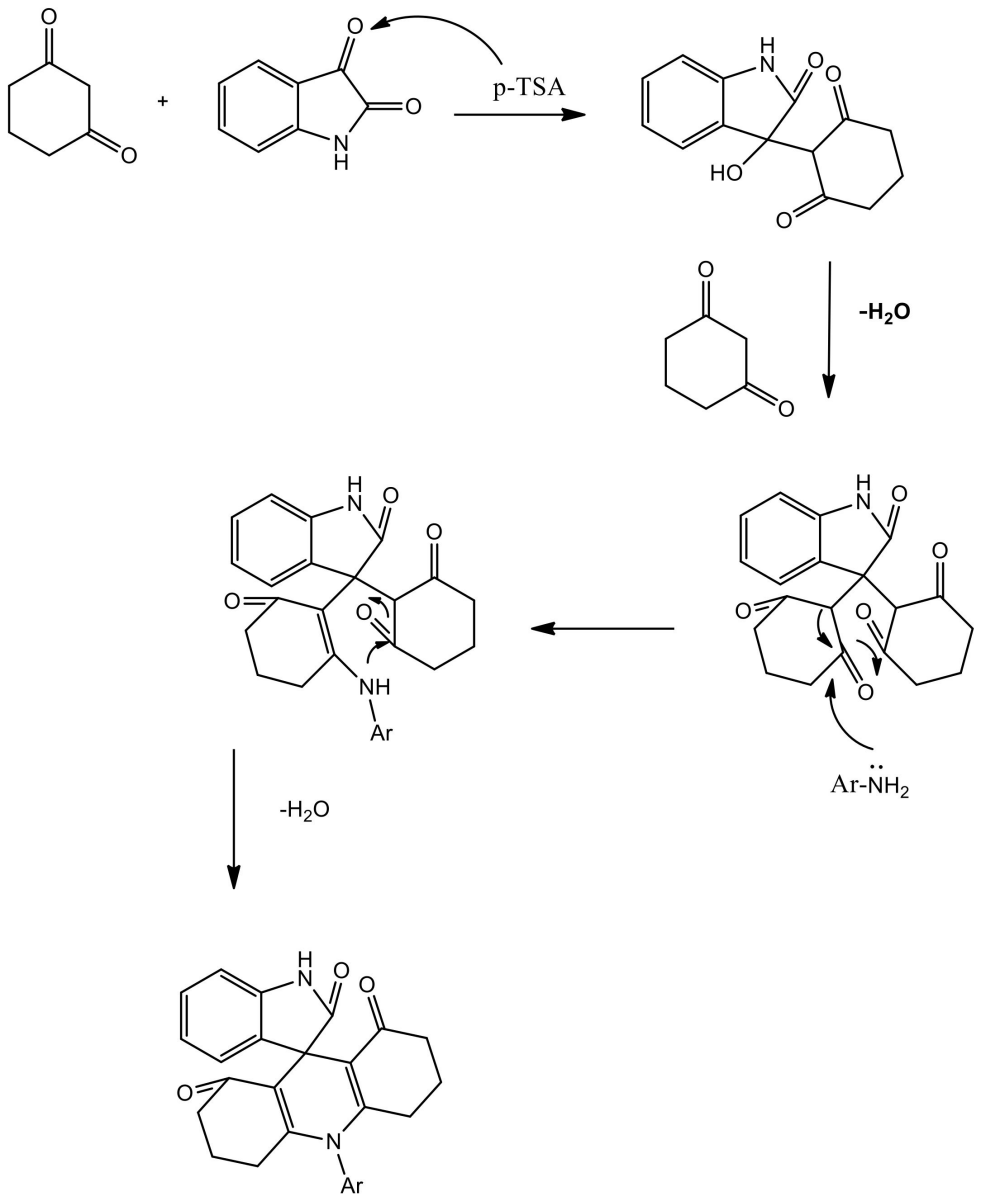
Proposed mechanism for the synthesis of spiro[acridine-9,2′-indoline]-1,3,8-trione.
