# Supplementary material for: Grindstone Chemistry: Design, One-Pot Synthesis, and Promising Anticancer Activity of Spiro[acridine-9,2′-indoline]-1,3,8-trione Derivatives against the MCF-7 Cancer Cell Line
Source: Molecules. 2020 Dec 11;25(24):5862. doi: 10.3390/molecules25245862 (PMC7763815; doi:10.3390/molecules25245862)

Supporting Information

Grindstone chemistry: Design, one-pot synthesis and promising anticancer activity of spiro[acridine-9,2′-indoline]-1,3,8-trione derivatives against MCF-7 cancer cell line

PerumalGobinath[1], PonnusamyPackialakshmi[1], Daoud Ali[2],Saud alarifi[2], Akbar Idhayadhulla[1], and SurendrakumarRadhakrishnan [1*]

Content

^{1}H-NMR Spectrum of compound- 2-11

^{13}C-NMR Spectrum of the compound 12-21

^{1}H-NMR

Compound **1a**

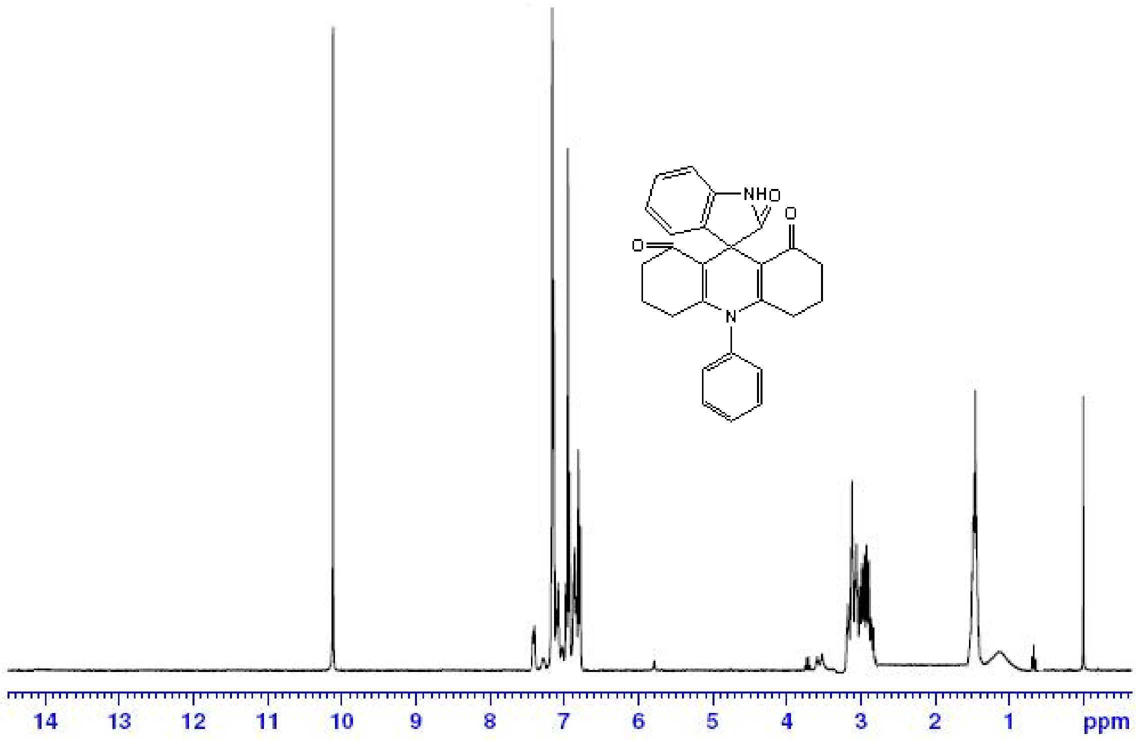

Compound **1b**

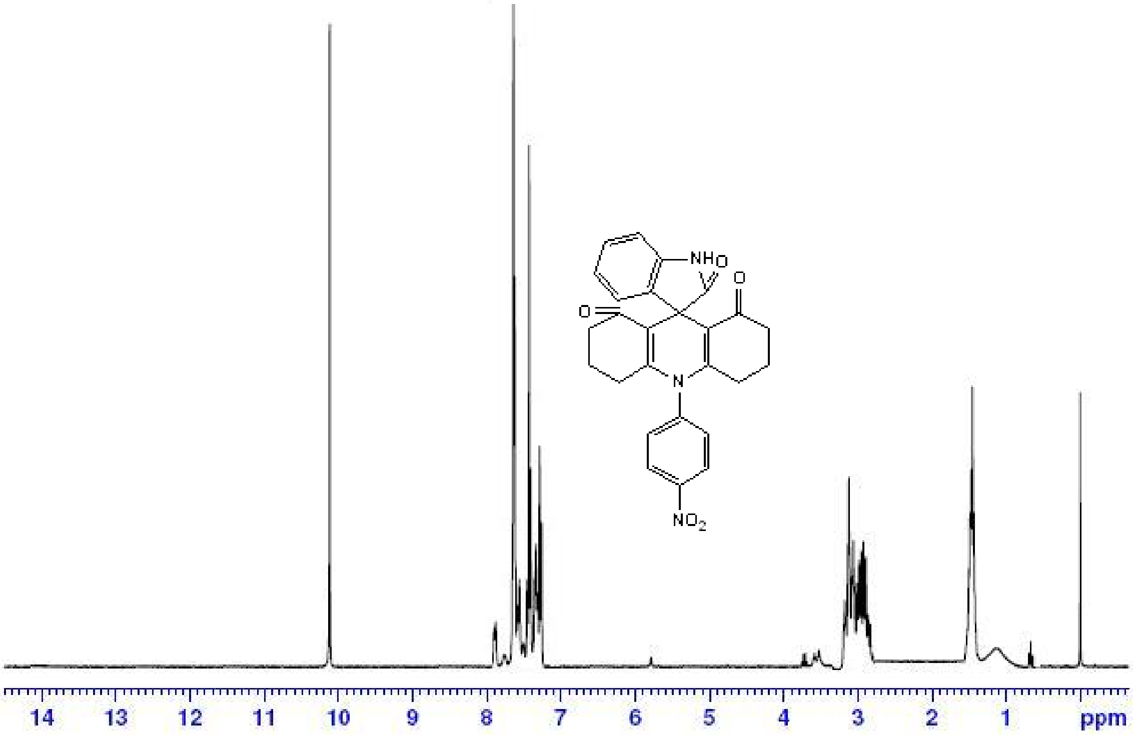

Compound **1c**

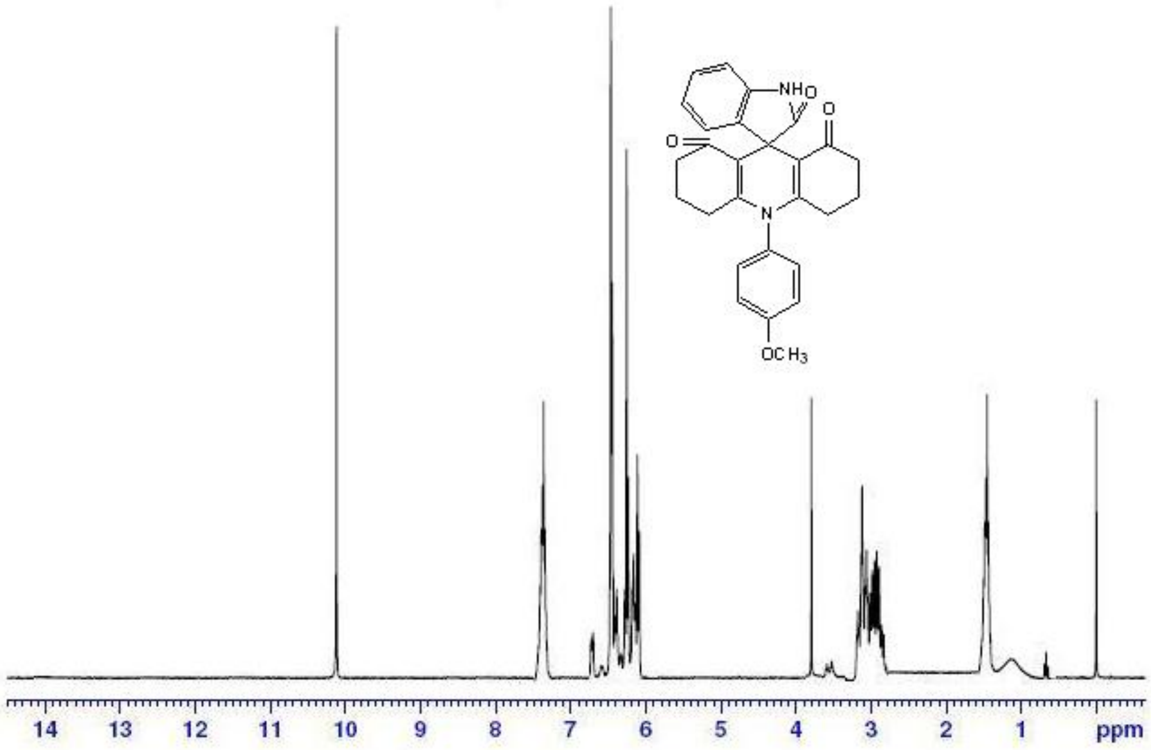

Compound **1d**

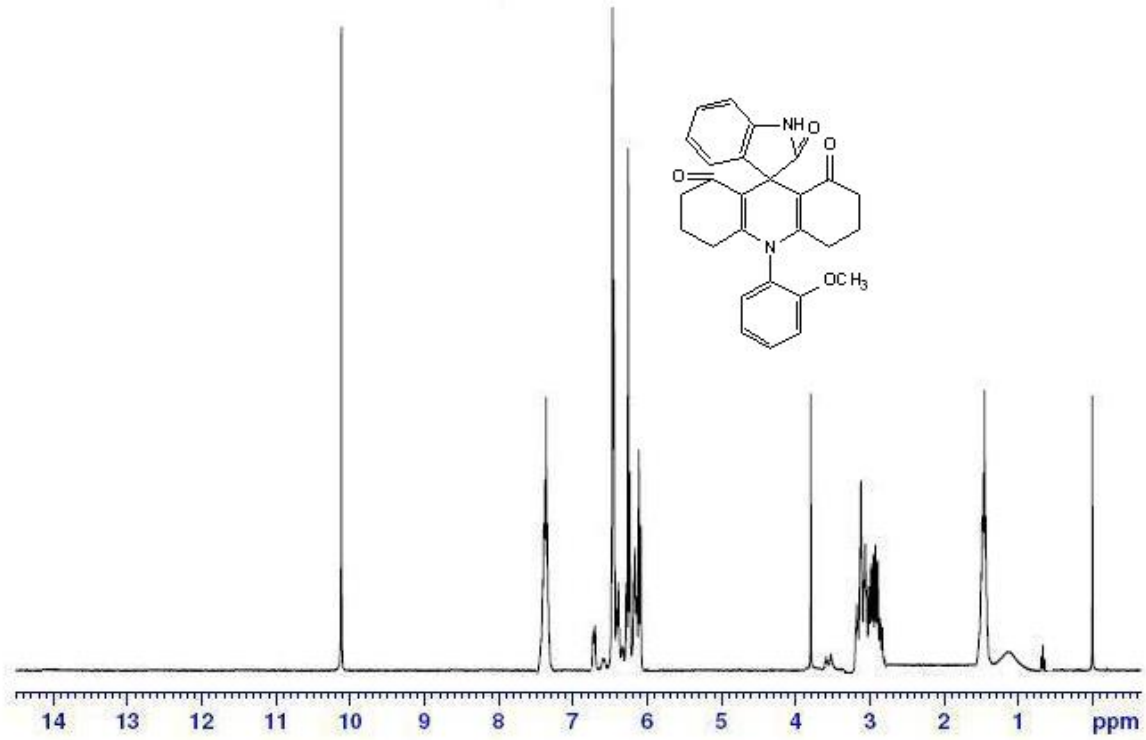

Compound **1e**

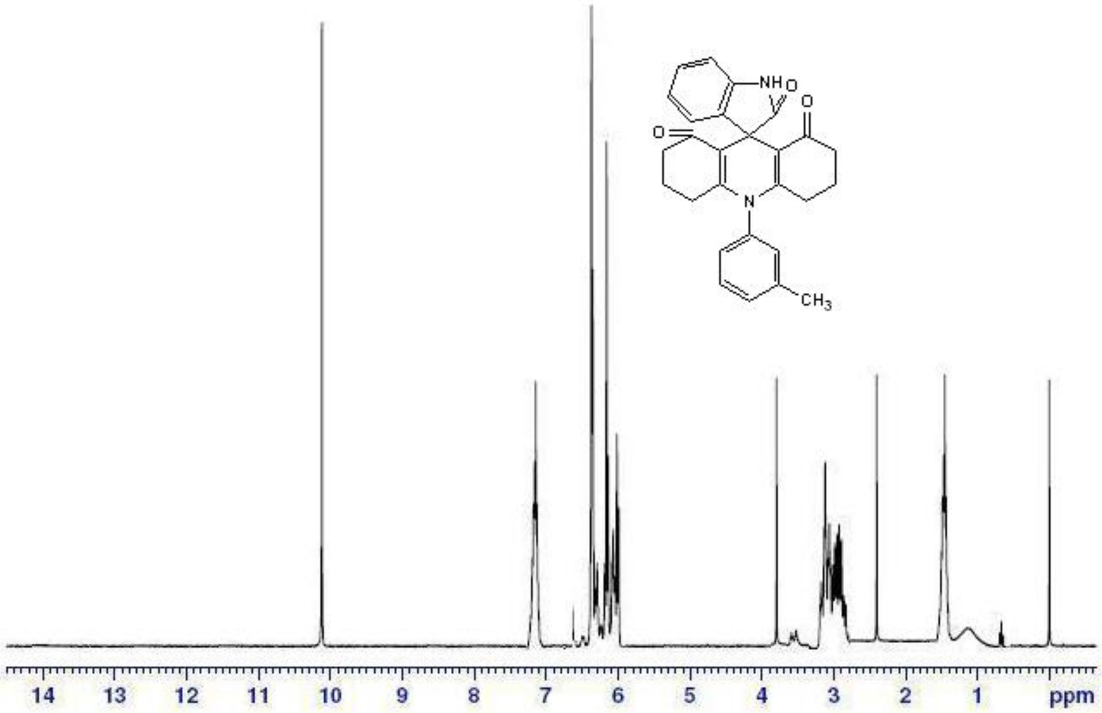

Compound **1f**

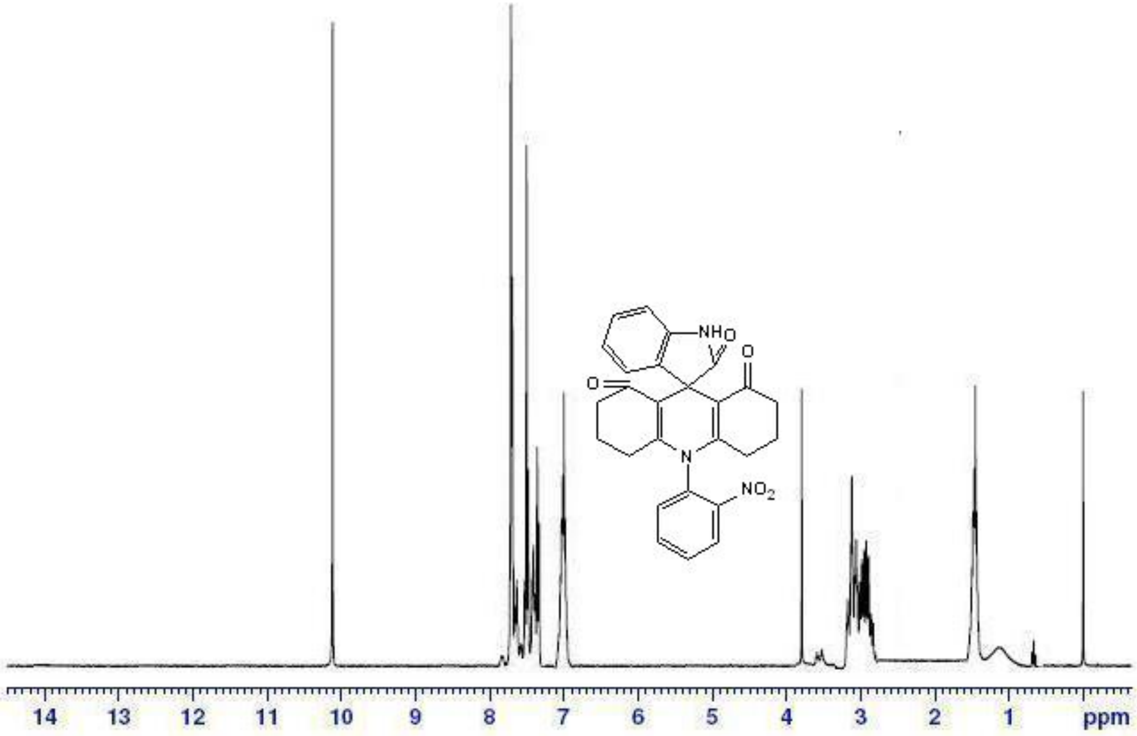

Compound **1g**

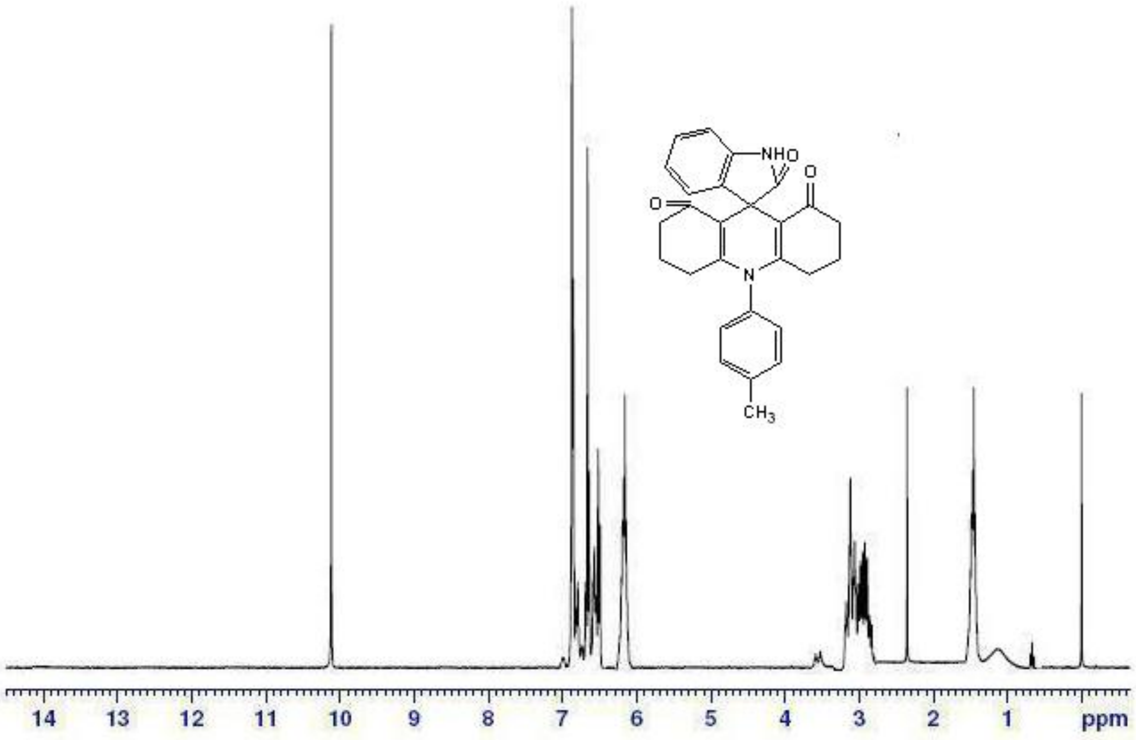

Compound **1h**

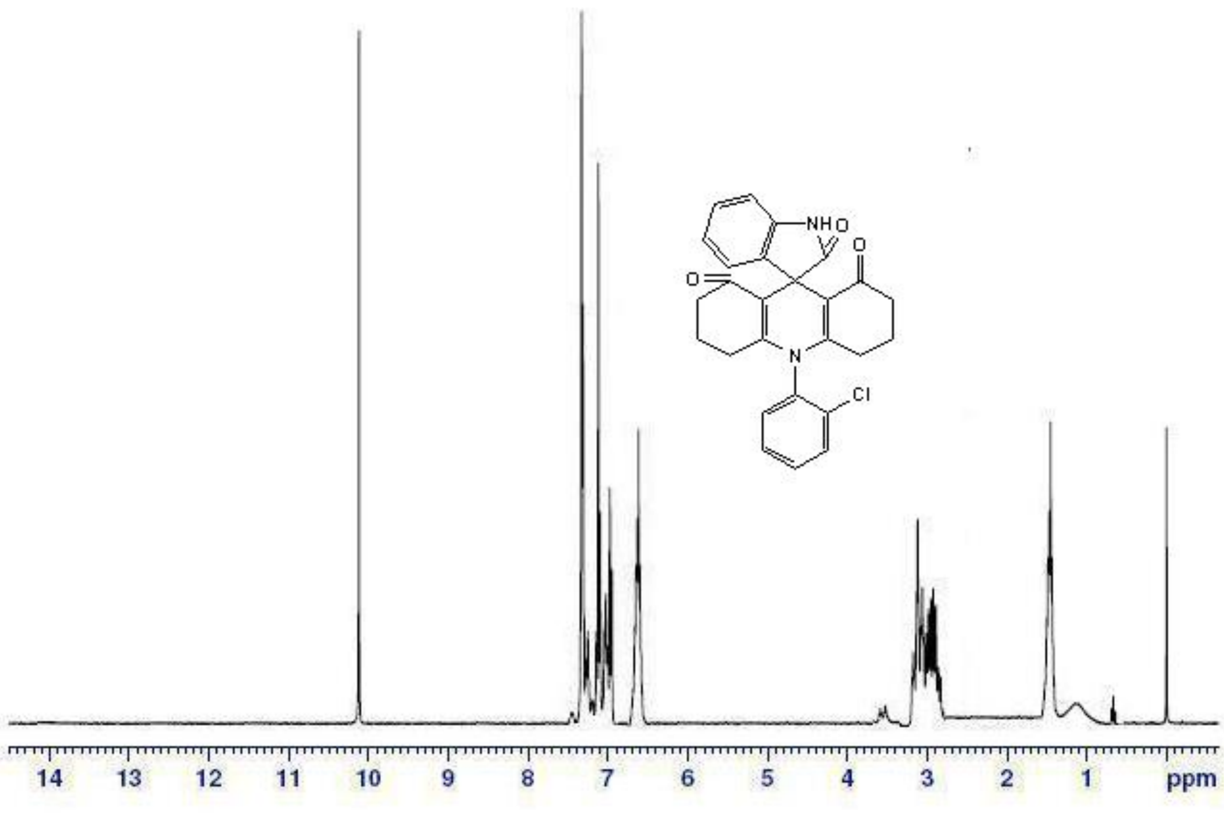

Compound **1i**

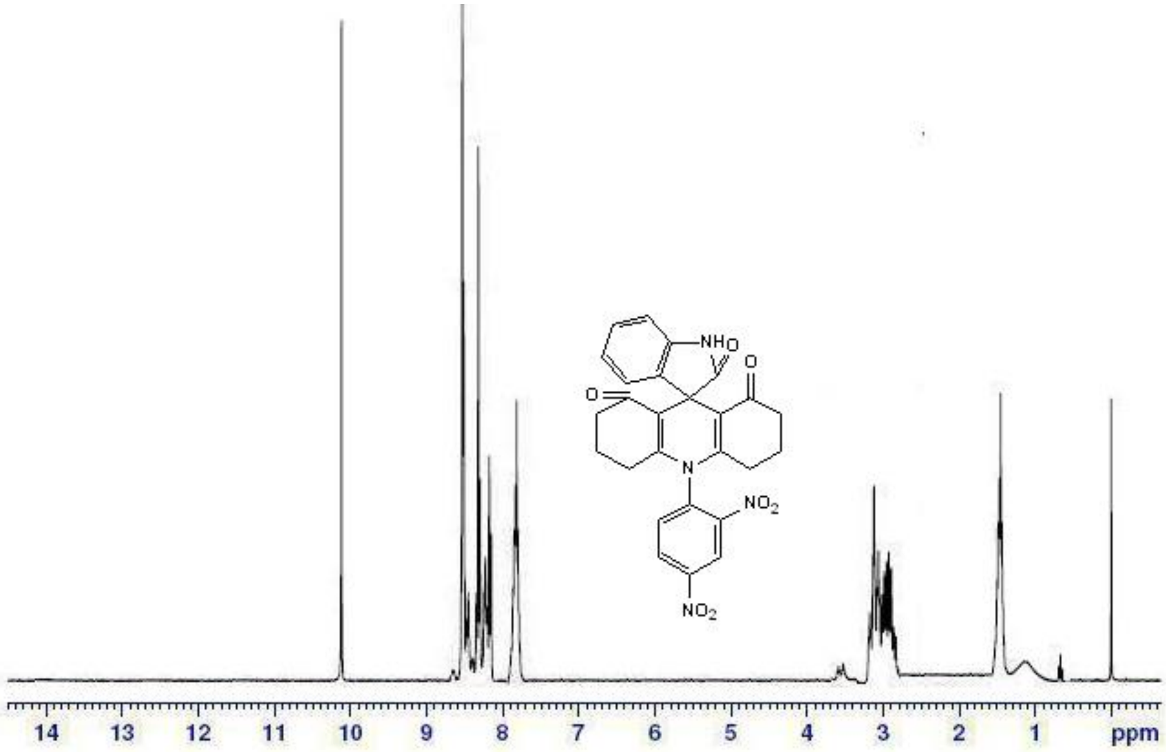

Compound **1j**

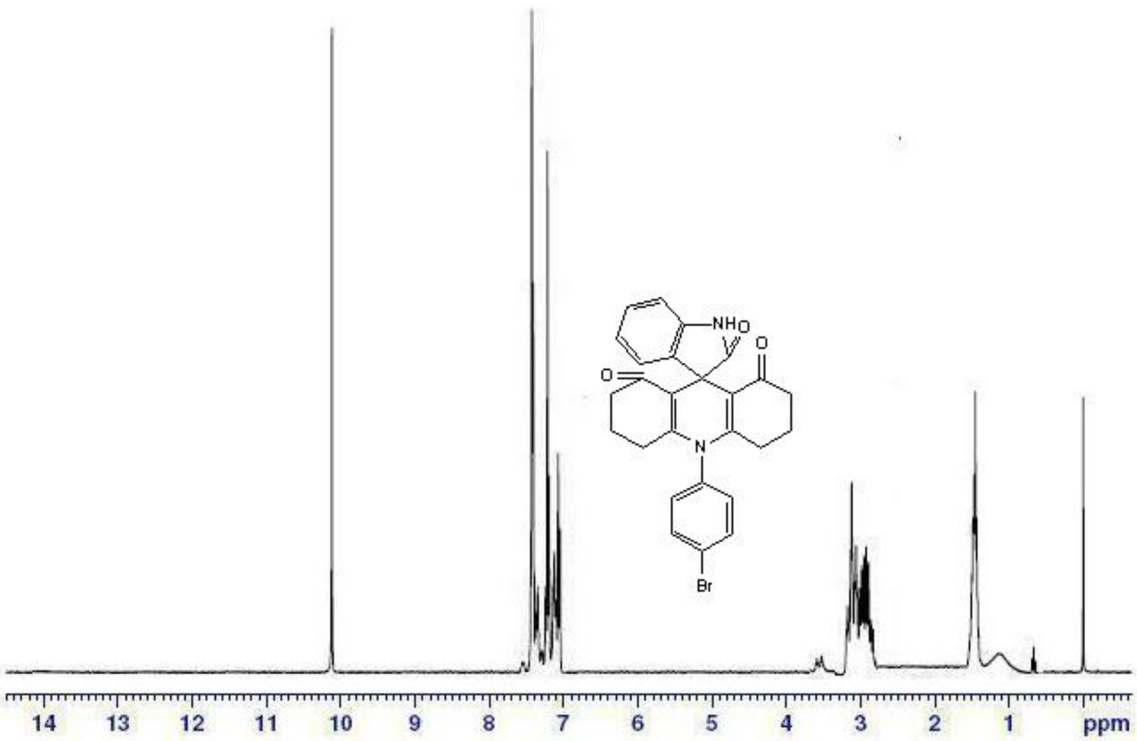

^{13}C-NMR

Compound-1a

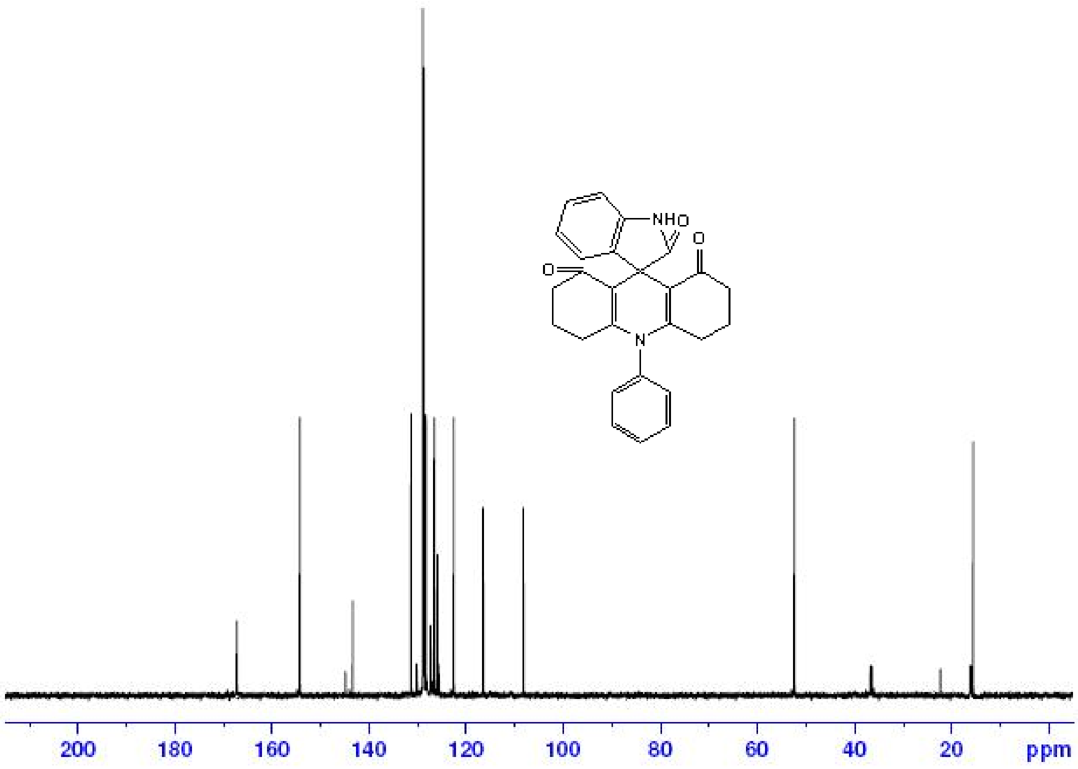

^{13}C-NMR

Compound-1b

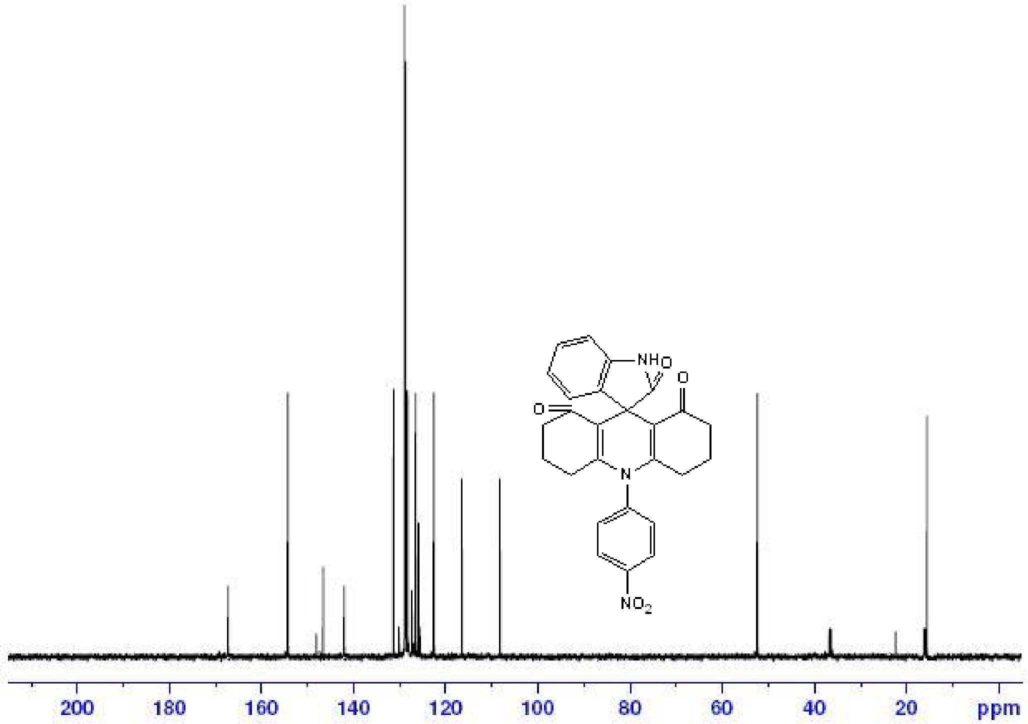

^{13}C-NMR

Compound-1c

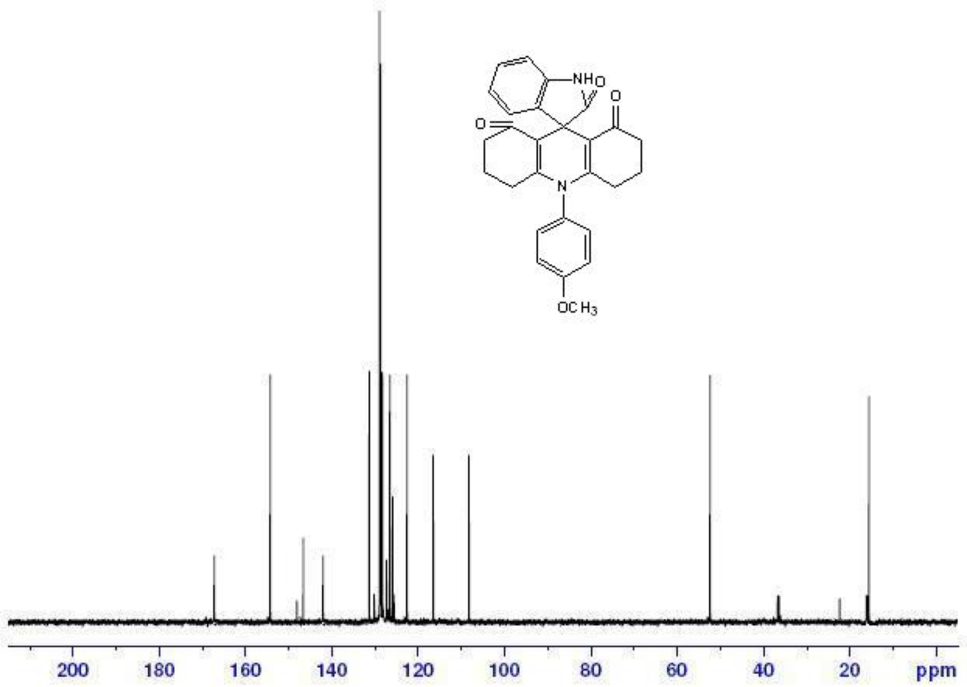

13-C-NMR

Compound-1d

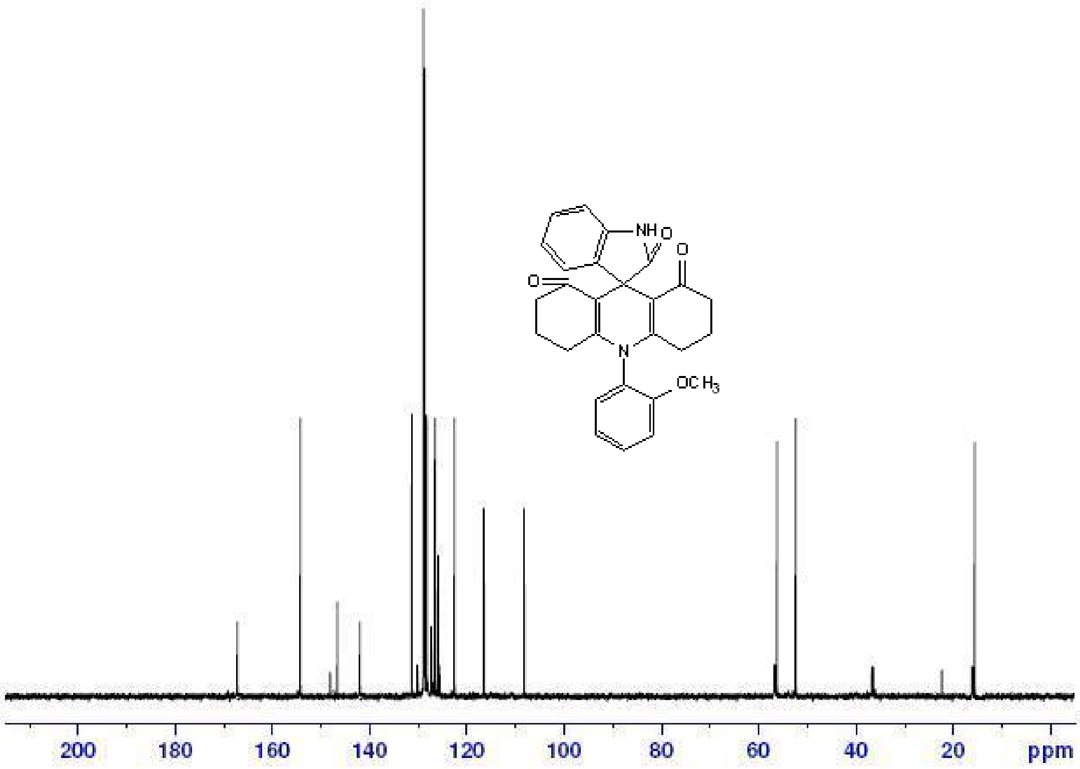

^{13}C-NMR

Compound-1e

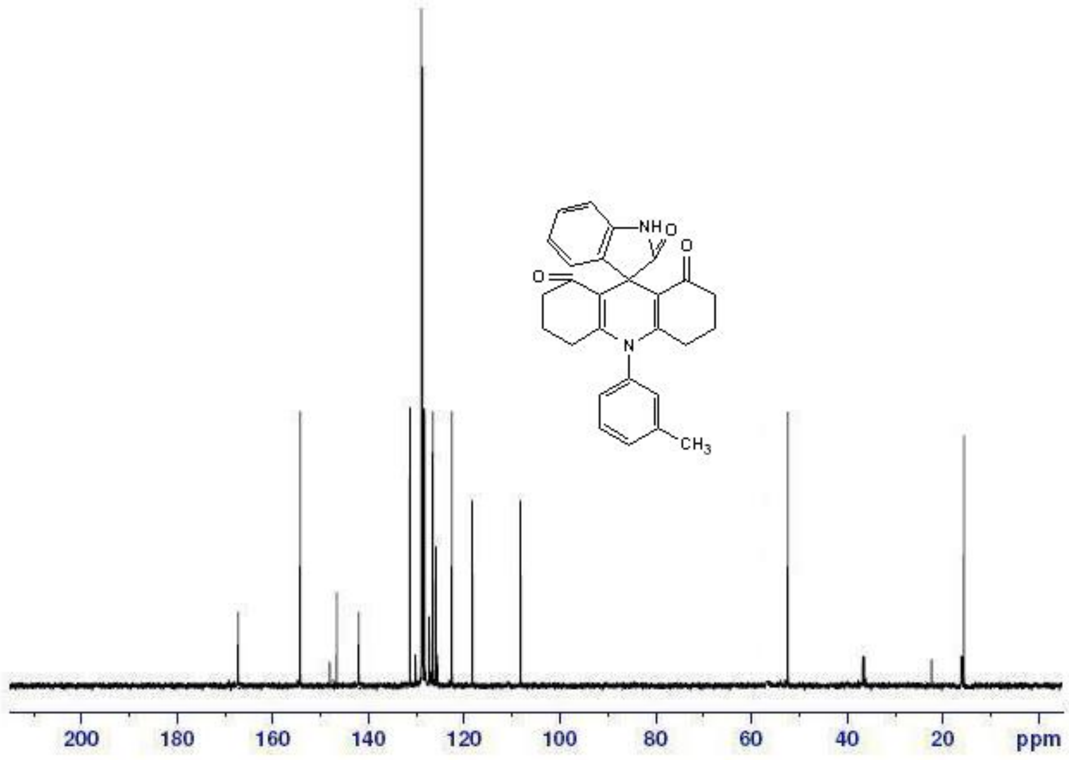

^{13}C-NMR

Compound-1f

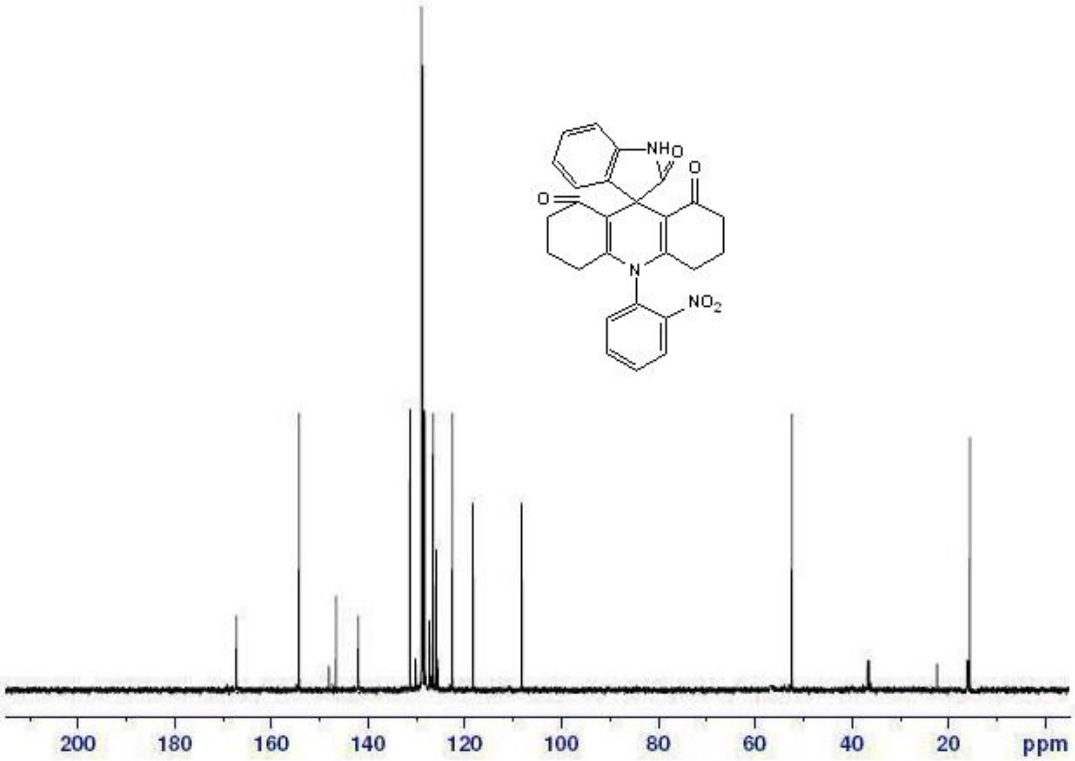

13-C-NMR

Compound-1g

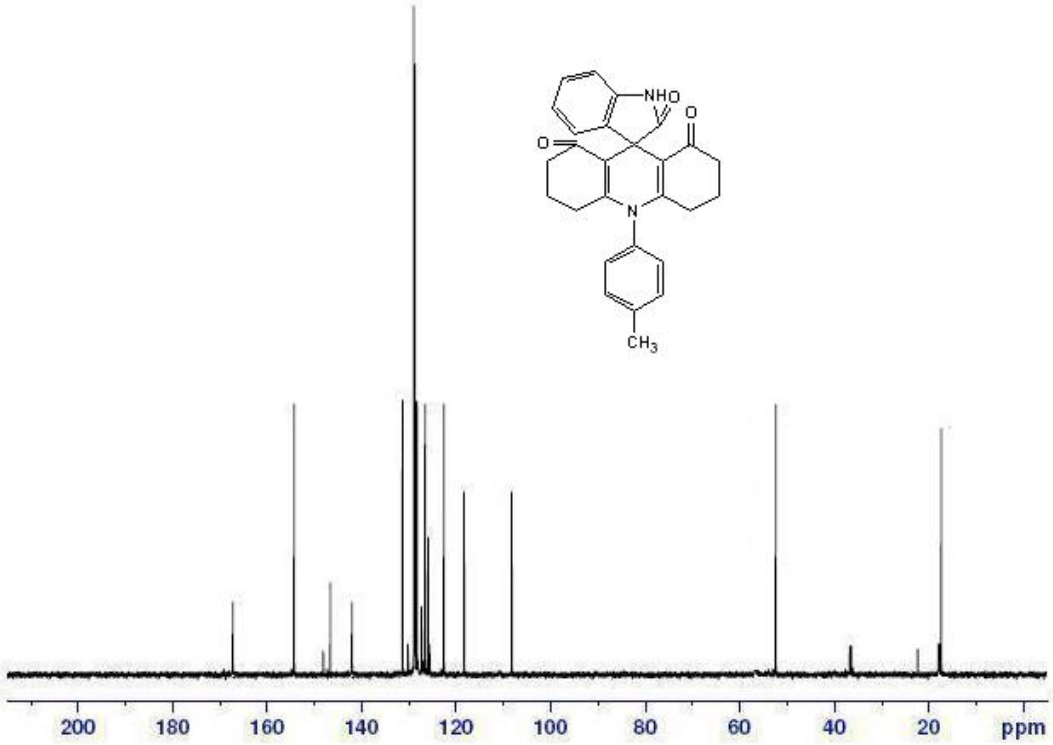

^{13}C-NMR

Compound-1h

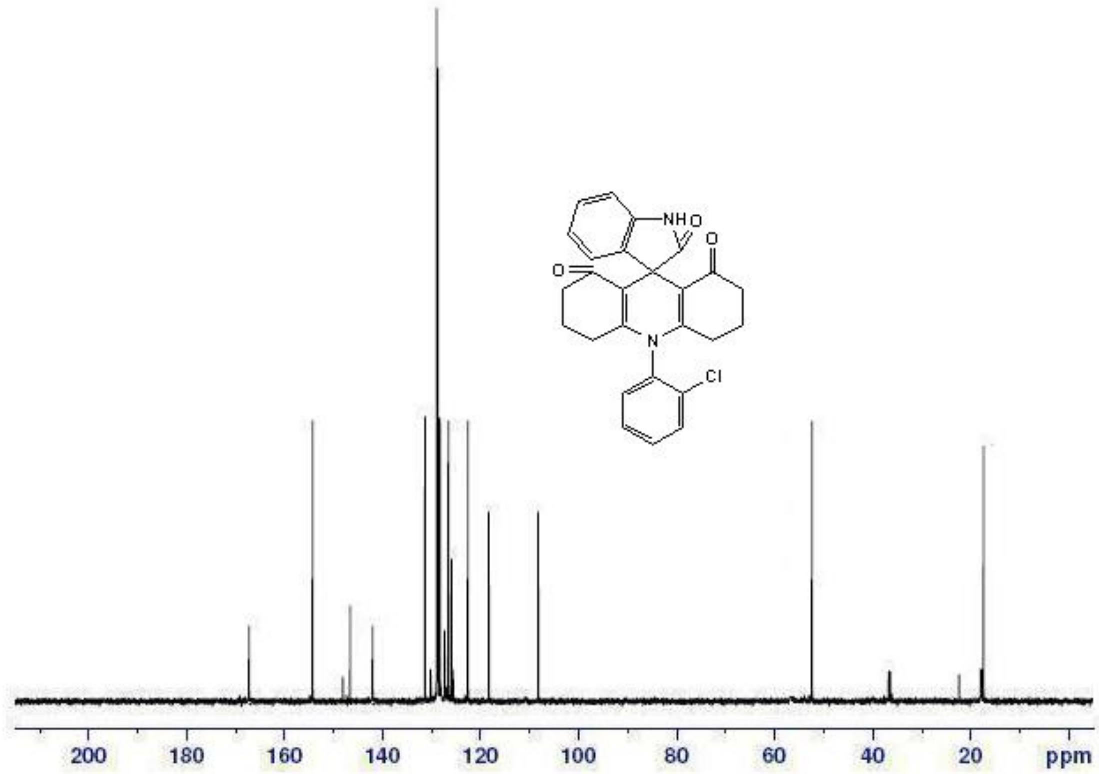

13-C-NMR

Compound-1i

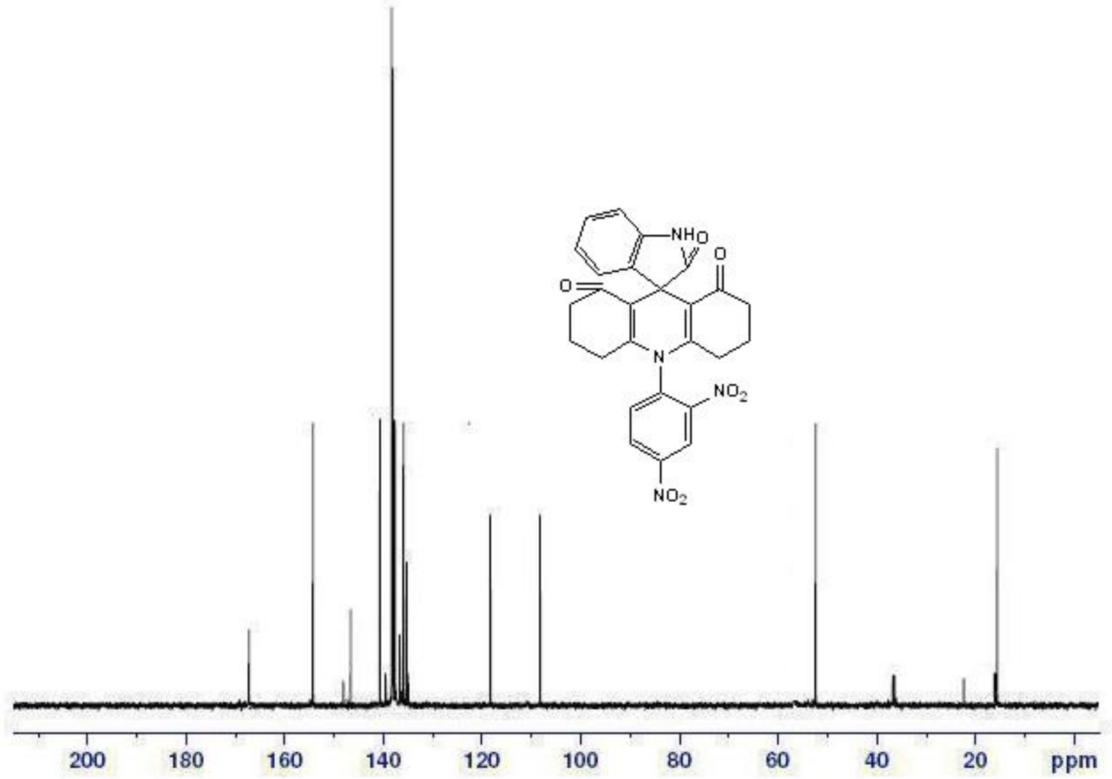

$^{13-}$C-NMR

Compound-1j

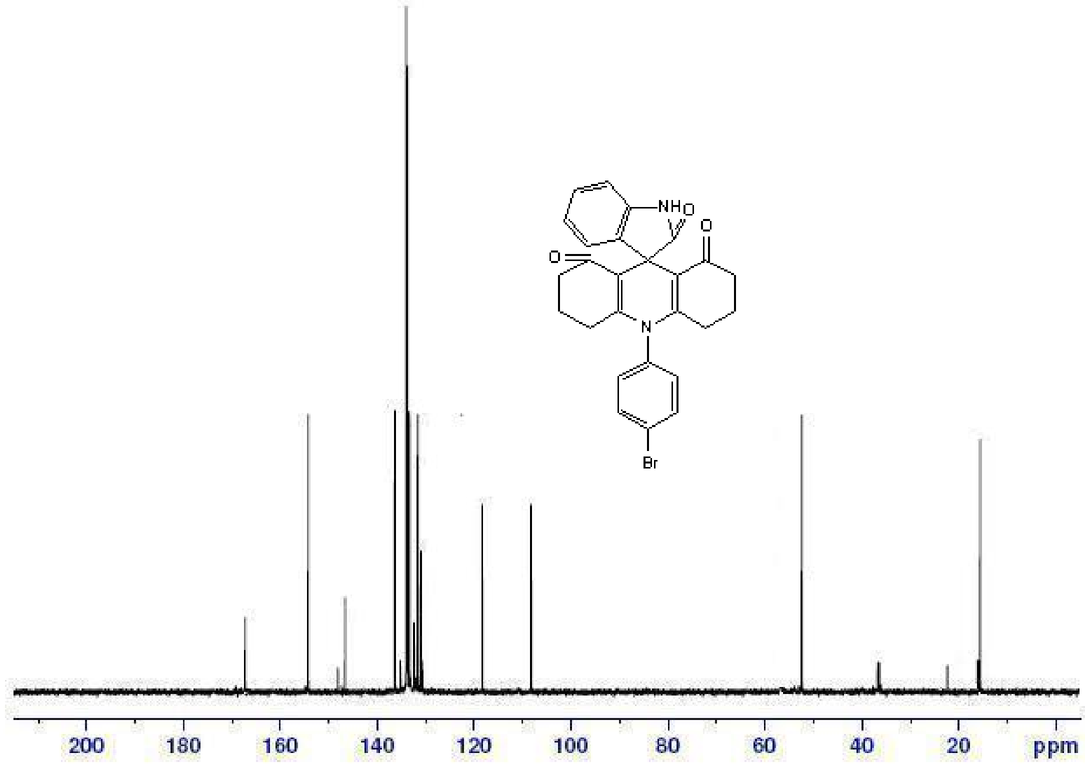

Supplement: Supplementary file 1 [file molecules-25-05862-s001.pdf]
